# Spatial, temporal, and molecular heterogeneity of ADC targets in high-grade serous ovarian carcinoma

**DOI:** 10.1038/s41416-026-03482-2

**Published:** 2026-06-02

**Authors:** Xiaoxuan Li, Tobias Janik, Markus Möbs, Stefan Florian, Wolfgang D. Schmitt, Amra Dzakulic, Jalid Sehouli, David Horst, Frank P. B. Dubois, Elena Ioana Braicu, Mihnea P. Dragomir

**Affiliations:** 1 Charité—Universitätsmedizin Berlin, Corporate Member of Freie Universität Berlin and Humboldt-Universität zu Berlin, Institute of Pathology, Berlin, Germany; 2 German Cancer Consortium (DKTK), Partner Site Berlin, and German Cancer Research Center (DKFZ), Heidelberg, Germany; 3 Charité—Universitätsmedizin Berlin, corporate member of Freie Universität Berlin and Humboldt-Universität zu Berlin, Department for Gynecology with the Center for Oncologic Surgery Charité Campus Virchow Klinikum, Berlin, Germany; 4 North Eastern German Society for Gynecological Cancer, Tumor Bank Ovarian Cancer Network, Berlin, Germany; 5 Berlin Institute of Health (BIH), Berlin, Germany

**Keywords:** Ovarian cancer, Tumour biomarkers

## Abstract

**Background:**

Antibody-drug conjugates (ADCs) represent a promising therapeutic approach for high-grade serous ovarian carcinoma (HGSOC). Patient selection for ADC therapy depends on tumour target expression, making it essential to characterize molecular, spatial, and temporal heterogeneity.

**Methods:**

We analyzed two HGSOC tissue microarray cohorts: 100 genomically profiled cases (1565 cores) and 64 matched cases with paired adnexal (A), locally advanced (LA), and recurrent (R) samples (2395 cores). Associations between ADC target expression and molecular characteristics, sampling site, and survival were investigated.

**Results:**

ADC targets showed no significant associations with homologous recombination deficiency (HRD) or *TP53* mutation status; TROP2 was modestly lower in *BRCA1/2*-mutated tumours. Folate receptor-alpha (FolR1) showed notable spatial heterogeneity: 20.2% switched therapeutic-indication groups between centre and margin at A; 21.7% were reclassified between A and LA. Temporally, all markers showed ≥ 20% switching, reaching 38.4% for FolR1 between A and R. High FolR1 expression in A correlated with poorer survival, a pattern not observed in LA or R samples.

**Conclusions:**

ADC targets in HGSOC display limited molecular but significant spatial and temporal heterogeneity, with expression classifications varying by site and time. FolR1 expression in adnexal tumours associates with aggressive disease.

**Figure:**
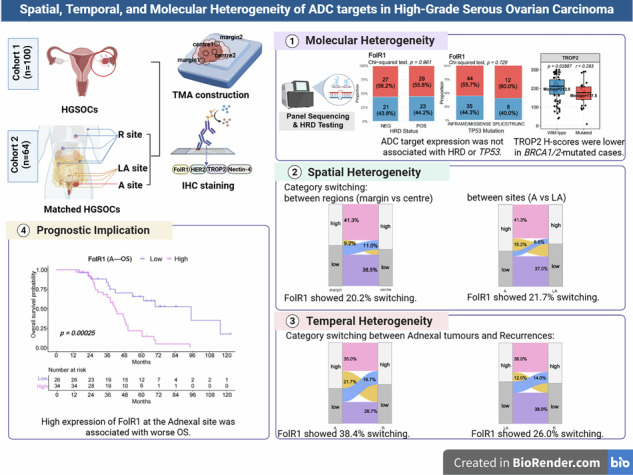

## Introduction

Ovarian cancer is the leading cause of death from gynecological malignancies worldwide. High-grade serous ovarian carcinoma (HGSOC) is the most common and aggressive type with poor prognosis [1]. Its high recurrence rate and treatment resistance are major problems in its clinical management [2]. One of the most characteristic molecular alterations in HGSOC is *TP53* mutation, present in nearly all cases [3–6]. In addition, HGSOC shows marked molecular heterogeneity. A major component of this heterogeneity is impaired DNA repair capacity, most notably homologous recombination deficiency (HRD), the loss of the homologous recombination repair (HRR) pathway for DNA double-strand break repair [7, 8]. About half of HGSOCs are HRD-positive, most commonly due to *BRCA1/2* mutations [9, 10]. This subgroup is particularly sensitive to platinum-based chemotherapy and PARP inhibitors, and HRD status is associated with improved overall survival (OS), even without PARP inhibition [11–17]. Beyond genomic alterations, HGSOC also exhibits spatial and temporal heterogeneity at the histological level, with site-specific variations observed between the primary adnexal tumour and peritoneal metastases [18, 19]. It is increasingly recognized that most HGSOCs likely arise from the Fallopian tube epithelium, particularly from serous tubal intraepithelial carcinoma (STIC) lesions, rather than the ovarian surface itself. Consequently, tumours traditionally referred to as “primary ovarian tumours” are more accurately described as adnexal tumours, a term that we have also adopted in this manuscript [20, 21].

Antibody–drug conjugates (ADCs) deliver cytotoxic agents to tumour cells via monoclonal antibodies. Folate receptor alpha (FRα/FolR1) is highly expressed in HGSOC but largely absent in normal tissues, making it an attractive therapeutic target [22–24]. Mirvetuximab soravtansine (MIRV), an ADC targeting FolR1, is EMA- and FDA-approved for platinum-resistant ovarian cancer, underscoring the clinical relevance of this strategy [22, 25]. Beyond FolR1, other antigens have emerged as promising targets for ADCs. Trophoblast cell-surface antigen 2 (TROP2) is frequently overexpressed in HGSOC and linked to poor prognosis, with the TROP2-targeted ADC datopotamab deruxtecan (Dato-DXd) showing potent preclinical and clinical efficacy [26–29]. HER2, although less common in HGSOC, correlates with aggressive tumour behavior; HER2-directed ADCs such as trastuzumab emtansine (T-DM1) and trastuzumab deruxtecan (T-DXd) have shown antitumour activity in preclinical ovarian cancer models, with the last one receiving tumour agnostic FDA approval in HER2 2 + /3+ expressing tumours, supported in part by the phase II DESTINY-PanTumor02 trial [30–32]. Nectin-4, a tumour-associated antigen highly expressed in cancer cells but largely absent in normal adult tissues, has been linked to adverse prognosis in ovarian cancers [33–39]. It represents a promising ADC target, exemplified by the approval of enfortumab vedotin for advanced urothelial carcinoma [40, 41]; however, its role in ovarian cancer remains unclear. The selective expression of FolR1, TROP2, HER2, and Nectin-4 highlights their therapeutic potential as ADC targets in HGSOC and provides a strong rationale for systematic evaluation. Importantly, patient eligibility is typically defined by immunohistochemistry (IHC) thresholds, such as FolR1-high status in MIRV trials and HER2 scoring in ongoing studies.

In addition to FolR1, HER2, TROP2, and Nectin-4, other ADC targets such as CDH6, B7-H4, and B7-H3 have also emerged in ovarian cancer, showing promising preclinical or early clinical activity [42, 43]. These developments further highlight the expanding therapeutic landscape of ADCs in HGSOC.

Although ADC targets show promise in ovarian cancer, most studies have focused on single antigens without systematic comparison under uniform conditions. Key issues remain unresolved, including their overall expression frequencies, inter-marker correlations, links to the molecular heterogeneity of HGSOC, and potential spatial or temporal variation across anatomical sites, tumour regions, or disease progression. Another gap is whether IHC-defined thresholds of ADC targets are associated with prognosis and reliably guide patient stratification. These limitations underscore the need for an integrated assessment of ADC target heterogeneity in HGSOC.

We hypothesize that ADC targets in HGSOC display molecular heterogeneity, interacting with genomic features such as HRD status, *TP53*, and *BRCA1/2* mutations to influence tumour biology and outcomes. We further hypothesize that ADC target expression also varies spatially and temporally, across adnexal and locally advanced sites, tumour regions, and between primary and recurrent diseases. Such heterogeneity may affect biomarker evaluation and patient stratification for ADC therapy.

## Materials and methods

### Study cohorts

**Fig. 1:**
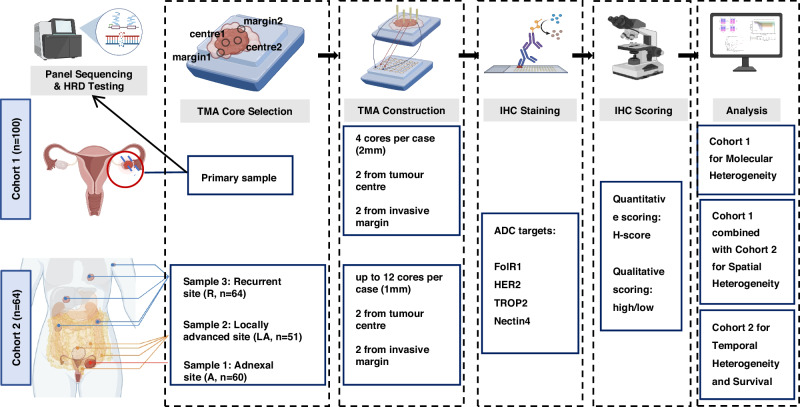
Overview of the study design, tissue microarray (TMA) construction, and the analytical workflow. TMA1 included 100 primary HGSOC cases, with four 2-mm cores per case sampled from the tumour centre and invasive margin. All cases underwent panel sequencing and HRD testing. TMA2 comprised 64 recurrent HGSOC cases, each with up to three tumour sites: adnexal tumour (Sample 1, A), locally advanced (Sample 2, LA), and recurrent tumour (Sample 3, R). Each site contributed four 1-mm cores, resulting in up to 12 cores per case. All samples were stained for four ADC targets (HER2, TROP2, Nectin-4, and FolR1) and evaluated using both H-score and high/low classification. The graphic elements were created on Biorender.com.

This study included two non-overlapping cohorts comprising 164 HGSOC cases who underwent surgery at Charité University Hospital Berlin or affiliated hospitals between 2014 and 2024 (Fig. 1). All tumours were histopathologically reviewed, classified according to the World Health Organization (WHO) Classification of Female Genital Tumours (5th Edition), and staged by the American Joint Committee on Cancer (AJCC) Cancer Staging Manual (8th Edition).

Cohort 1 (TMA1) consisted of 100 primary HGSOC cases reviewed and tested for homologous recombination deficiency (HRD) at the Institute of Pathology, Charité, between 2021 and 2024. HRD testing was performed using the NOGGO GIS v1 assay (details in Section 2.4). Cohort 2 (TMA2) comprised 64 HGSOC cases diagnosed between 2014 and 2021, all of which recurred between 2015 and 2023. Each patient underwent surgery and tumour sampling at both primary diagnosis and recurrence. For both cohorts, clinical and pathological data were collected, including age at diagnosis, T stage, FIGO stage, follow-up information, and detailed treatment history (Supplementary Table S1).

The conduct of this study was approved by the local ethics committee (EA1/110/22).

### Tissue microarray (TMA) construction

For both cohorts, formalin-fixed, paraffin-embedded (FFPE) tumour blocks were used to construct two independent TMAs. Lymphoid tissue was included on each array for orientation. To investigate potential intratumour heterogeneity, TMA cores were sampled from both the tumour centre and tumour margin regions. This approach was designed to capture possible micro-regional differences in tumour biology, including variations in nutrient availability, stromal composition, and immune microenvironment, which may influence ADC target expression. Within the same paraffin block, tumour centre was defined as tumour tissue surrounded by tumour cells on all sides and clearly separated from the tumour–stromal interface (minimum distance ≥ 2 high-power fields); tumour margin was defined as the tumour area located at the tumour–stromal interface (Supplementary Fig. S1A). The representative example of TMA cores from the centres and margins is shown in Supplementary Fig. S1B. For clarity and to reflect anatomical sampling sites, tumour specimens were categorized as follows: Tissue specimens obtained from the adnexal primary tumour site are referred to as adnexal tumour (A). Tissue specimens obtained from tumour dissemination confined to the pelvis or regional lymph nodes are referred to as locally advanced tumour (LA). Only a small number of LA samples were derived from lymph node metastases (three in Cohort 1 and one in Cohort 2). Tissue specimens obtained from recurrent tumours are referred to as recurrent tumour (R).

Cohort 1 (TMA1) comprised 100 primary HGSOC cases, each represented by four 2.0-mm cores: two from the tumour centre and two from the tumour margin. Based on anatomical sampling sites, 55 cases were classified as A and 45 as LA. In total, 1565 valid cores were available for analysis. Cohort 2 (TMA2) included 64 HGSOC patients with paired A (*n* = 60), LA (*n* = 51), and R (*n* = 64) specimens when available. From each site, four 1.0-mm cores were sampled (two centre and two margin). Of the 64 cases, 47 had cores from all three sites. Tissue loss occurred in one case during Nectin-4 and FolR1 IHC; four cases lacked A but had LA, and 13 lacked LA but retained A. In total, 2395 valid cores were available from TMA2. Altogether, 3960 evaluable tissue cores were included in the study. The analytical workflow, including TMA construction, IHC staining, scoring, and statistical analysis, is illustrated in Fig. 1.

### Immunohistochemistry (IHC) and scoring

For IHC, TMA blocks were cut into 2-µm sections and incubated in CC1 buffer (Ventana) according to protocol (CC1 mild for HER2/Nectin-4, CC1 standard for FolR1, 16-min CC1 for TROP2). Primary antibodies were anti-HER2 (clone 4B5, Ventana, RTU), anti-Nectin-4 (clone EPR15613-68, Abcam, 1:100), anti-TROP2 (clone 01, Enzo, 1:500), and anti-FolR1 (clone FOLR1-2.1, Roche, RTU). Incubation was 32 min at room temperature, with visualization by the avidin-biotin complex method and 3,3′-diaminobenzidine. Staining was performed on the BenchMark Ultra platform (Ventana). Nuclei were counterstained with hematoxylin/bluing reagent (Ventana) for 12 min. Slides were evaluated using Olympus BX50/BX46 microscopes, and whole-slide images were acquired with a high-resolution digital scanner for pathological review.

IHC staining was primarily assessed by a certified pathologist (X.L.); cases with uncertain scores were independently reviewed by two other pathologists (F.D. and M.P.D.), and results were reached by consensus. For ADC-target markers, staining intensity was graded as 0 (negative), 1+ (weak), 2+ (moderate), or 3+ (strong). The percentage of tumour cells at each level was recorded, and an H-score was calculated: H-score = (% of 1+ cells) × 1 + (% of 2+ cells) × 2 + (% of 3+ cells) × 3, ranging from 0–300.

**Fig. 2:**
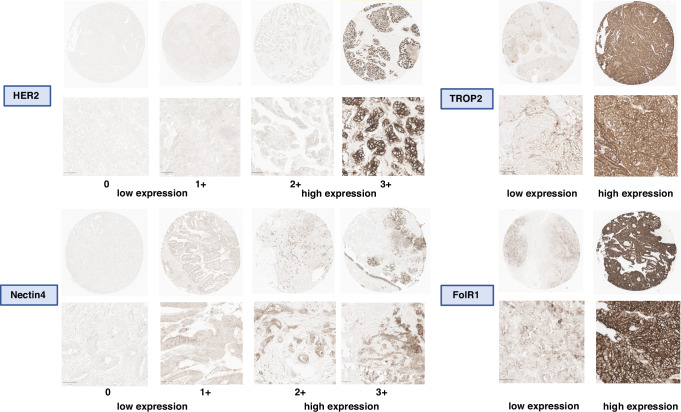
Representative IHC expression patterns and scoring criteria for ADC targets. HER2 and Nectin-4 were scored using a 4-tier system (0, 1+, 2+, 3+); for binary classification, scores of 0 and 1+ were considered low expression, and 2+ and 3+ as high expression, as defined in the “Methods” section. TROP2 and FolR1 were assessed using a binary classification system (low vs. high).

For ADC marker classification, binary thresholds were applied based on previous studies or clinical trial data. HER2 expression was evaluated according to the American Society of Clinical Oncology and the College of American Pathologists (ASCO/CAP) guidelines for gastroesophageal adenocarcinoma [44]. A score of 3+ was defined as strong membranous staining, either complete or basolateral, in ≥ 10% of tumour cells; 2+ indicated moderate to weak staining in ≥ 10% of cells; 1+ weak or incomplete staining in ≥ 10%; and 0 no staining or < 10% of cells. For statistical purposes, HER2 scores of 2+ and 3+ were defined as high expression, while 0 and 1+ were defined as low expression [32]. For TROP2, we used an H-score cutoff of 200, as reported in recent OC/EC studies [28]. H-score ≥ 200 defined high, < 200 low. FolR1 high expression was defined as ≥ 75% of tumour cells with moderate/strong staining [45]. Nectin-4 was classified as 0 (H-score 0–14), 1+ (15–99), 2+ (100–199), and 3+ (200–300) [46]; H-scores ≥ 100 were considered high, < 100 low. Representative IHC staining patterns and scoring criteria for each ADC target are illustrated in Fig. 2.

### Molecular profiling (Cohort 1)

Homologous recombination deficiency (HRD) was assessed with the NOGGO GIS v1 assay [47], a hybrid-capture NGS panel covering *BRCA1/2*, 55 HRR genes, additional driver mutations (*TP53*, *KRAS*, *NRAS*, *PIK3CA*, *BRAF*), and copy number alterations. Genomic instability (GI) was measured by three parameters: percentage of loss of heterozygosity (PLOH), copy number alterations (PCNA), and telomeric copy number alterations (PTCNA), which were combined to generate a genomic instability score (GIS). HRD positivity was defined as either a GIS ≥ 83 or the presence of pathogenic *BRCA1/2* mutations. *TP53* mutations were classified as missense, in-frame indels, splice-site alterations, or truncating mutations (including nonsense and frameshift). For *TP53*, missense/in-frame variants were analyzed together because they usually produce full-length p53 with dominant-negative or gain-of-function properties. In contrast, splice-site and truncating variants disrupt canonical splicing or introduce premature stop codons, which typically trigger nonsense-mediated decay or produce truncated proteins lacking critical domains, resulting in loss of function [48]. We therefore combined variants by their expected protein consequence to increase statistical power while reflecting mechanistic similarity.

### Statistical analysis

Analyses were performed in R (v4.4.2). All tests were two-sided, with *p* < 0.05 considered significant.

#### Molecular heterogeneity analysis in Cohort 1

To evaluate associations between ADC target expression and HRD, *TP53*, and *BRCA1/2* status, we compared quantitative H-scores (non-normal by Shapiro–Wilk) using Wilcoxon rank-sum tests and high/low categories using *χ*² or Fisher’s exact tests. For *TP53*, missense and in-frame variants were grouped, and splice-site variants with truncating variants, reflecting functional similarities. For *BRCA1/2* as mutated versus wild type.

#### Spatial heterogeneity analysis across two cohorts

To improve clarity, centre–margin comparisons were subsequently combined across cohorts and stratified by anatomical tumour site (A, LA, R) rather than cohort origin. For this purpose, samples from Cohort 1 (*n* = 100) were split into A (*n* = 55) or LA (*n* = 45) according to their anatomical sampling site. We compared tumour margin versus tumour centre at A, LA, and R. With two cores per region, we used the mean H-score and assigned the higher category if categorical calls differed; single cores were used as is. Pairs require 1 margin and ≥1 centre core. Wilcoxon signed-rank tested H-scores; McNemar tested high/low classifications. We also assessed A versus LA pairs using the same methods.

#### Temporal heterogeneity analysis in Cohort 2

We assessed temporal heterogeneity of ADC targets in Cohort 2 by comparing expression between paired primary and recurrent tumours (A–R and LA–R). H-scores were analyzed with Wilcoxon signed-rank tests; categorical high/low with McNemar tests. Sankey plots visualized category changes. The switching rate between groups was defined as the proportion of cases that changed category (low-high) from A/LA to R.

To evaluate whether systemic therapy exposure influenced changes in ADC target expression, we analysed categorical transitions between A and R samples (high-to-low, low-to-high, or stable). Patients were stratified according to bevacizumab exposure, PARPi treatment, and number of chemotherapy cycles received between initial and recurrence surgeries (Supplementary Table S2). Transition patterns were visualized using stacked bar plots, and associations between treatment exposure and expression changes were assessed using Fisher’s exact tests.

#### Survival analysis in Cohort 2

Survival analysis was conducted in Cohort 2, which had longitudinal follow-up data for all patients (median follow-up 41.7 months, interquartile range 28.8–63.7 months; Supplementary Table S1). In contrast, follow-up in Cohort 1 was complete for 93/100 cases and was substantially shorter overall (median follow-up 17.3 months, interquartile range 11.1–21.5 months). OS was defined as the interval from the date of primary diagnosis to death or last follow-up. Progression-free survival (PFS) was defined as the interval from the date of primary diagnosis to radiologically or clinically confirmed progression or death, whichever occurred first. We assessed the prognostic value of ADC targets by Kaplan–Meier curves: OS and PFS were analyzed in A/LA tumours, with log-rank tests comparing differences between high- and low-expression groups. Associations with survival were further tested using univariable Cox models; significant markers were entered into multivariable models with clinical covariates. Age, T-stage, and platinum sensitivity were selected as covariates, as they are established prognostic factors in HGSOC. Platinum sensitivity was defined by the platinum-free interval (PFI): < 6 months resistant, 6–12 months partially sensitive, and >12 months sensitive [49, 50]. Restricting the model to these variables reduces the risk of overfitting and ensures that the independent prognostic value of ADC marker expression is evaluated within the context of the most relevant clinical parameters.

## Results

### Most HGSOCs show expression of multiple ADC targets without any correlation to known molecular subtypes

To test our hypothesis that ADC target expression reflects molecular heterogeneity in HGSOC and interacts with genomic features such as *HRD*, *TP53*, and *BRCA1/2*, we analyzed 100 genomically well-characterized primary tumours.

**Fig. 3:**
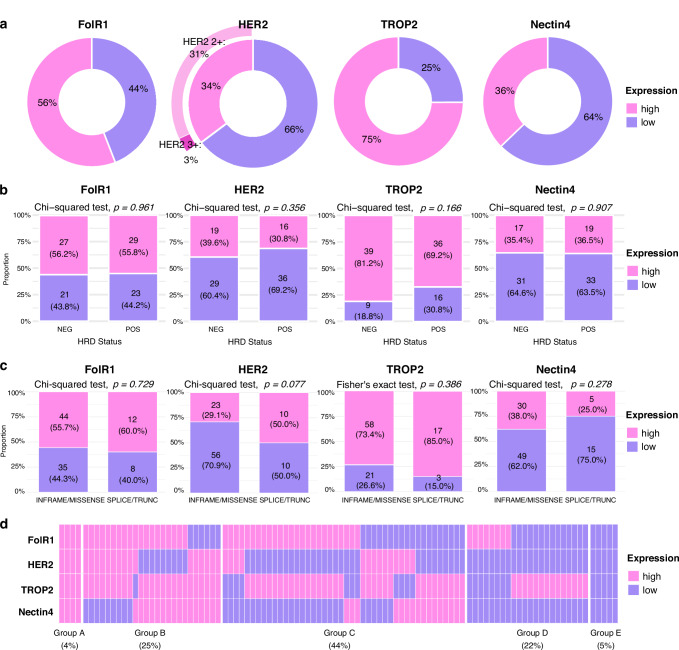
Limited molecular ADC heterogeneity in Cohort 1. **a** Distribution of high and low expression levels for FolR1, HER2, TROP2, and Nectin4 across Cohort 1. **b** Proportion of ADC target expression groups stratified by HRD status. **c** Proportion of ADC target expression groups stratified by *TP53* mutation type (in-frame/missense vs splice-site/truncating). **d** Co-expression groups based on the number of high-expressed ADC targets (Group A: all targets high expression; Group B: 3 targets high expression; Group C: 2 targets high expression; Group D: 1 target high expression; Group E: all targets low expression). **Abbreviations**: HRD homologous recombination deficiency, NEG negative, POS positive.

Across this cohort (*n* = 100; 1565 evaluable TMA1 cores), ADC marker expression varied widely. TROP2 (75%) and FolR1 (56%) were most frequently overexpressed, whereas HER2 (34%) and Nectin-4 (36%) showed lower prevalence. Among HER2-high cases, most (31/34) exhibited 2+ intensity, while only a minority (3/34) reached 3+ intensity (Fig. 3a). We assessed whether ADC target expression differed by HRD status. Quantitative H-scores did not significantly differ between HRD-positive and HRD-negative tumours (all *P* > 0.05, Wilcoxon test; Supplementary Fig. S2A). Likewise, the prevalence of high versus low expression showed no significant association with HRD (all *P* > 0.05, Chi-square test; Fig. 3b).

When analysed as a continuous variable (H-score), TROP2 expression was modestly lower in *BRCA1/2*-mutated than in wild-type tumours (Wilcoxon *p* = 0.029), whereas FolR1, HER2, and Nectin-4 showed no clear differences (Supplementary Fig. S2B). By *TP53* mutation type, none of the four markers differed in H-score (Supplementary Fig. S2C).

Given that ADC-based therapeutic eligibility is commonly defined using categorical biomarker thresholds, expression was dichotomised into high and low categories. High-expression rates were broadly similar between *BRCA1/2*-mutated and wild-type tumours (HER2 34.7% vs 32.0%, TROP2 77.3% vs 68.0%, Nectin-4 34.7% vs 40.0%, FolR1 52.0% vs 68.0%), with no significant differences (Supplementary Fig. S3). A borderline association was observed for HER2 by *TP53* mutation type (*χ*² = 3.80, *p* = 0.077), with high expression more frequent in splice-site/truncating than in in-frame/missense *TP53* mutations (Fig. 3c).

Next, we investigated whether the four ADC targets were co-expressed within individual tumours. Spearman correlation analysis of H-scores showed only a weak positive correlation between HER2 and Nectin-4 (*ρ* = 0.20, *p* = 0.044) (Supplementary Fig. S4). We next summarized tumours by the number of high-expression targets. In Cohort 1, 5% of cases were antigen-negative (0/4 high); 22% showed a single target only, whereas 73% showed ≥ 2 high-expressed targets (Fig. 3d).

### ADC targets show site-specific heterogeneity leading to discrepant eligibility scores

In Cohort 2 (*n* = 64; 2395 evaluable TMA2 cores), a similar pattern of ADC expression was observed in adnexal tumours (A): TROP2 (85%) and FolR1 (57%) remained the most prevalent high-expression targets, whereas Nectin-4 (37%) and HER2 (20%) were less common. To test our hypothesis that metabolic or immune responses may drive microscopic spatial heterogeneity within individual tumours, ADC target expression was analysed in the combined cohort. Across the combined cohort, ADC target expression was evaluated separately at A, LA, and R by comparing expression between tumour centre and margin.

First, centre–margin differences were evaluated using paired H-scores. In A tumours, HER2 showed a higher level in the tumour margin compared to the centre, with a *p* value approaching statistical significance but with a negligible effect size (*p* = 0.058, *r* = 0.000). FolR1, TROP2, and Nectin-4 showed no statistically significant differences between centre and margin. In LA tumours, HER2 (*p* = 0.012, *r* = −0.265) and Nectin-4 (*p* = 0.003, *r* = −0.316) showed statistically significant but modest differences, with higher H-scores in tumour margins than in tumour centres, whereas FolR1 (*p* = 0.643, *r* = 0.000) and TROP2 (*p* = 0.702, *r* = 0.000) did not. In R tumours, no significant margin–centre differences were detected (all *p* > 0.2) (Supplementary Fig. S5A–C).

**Fig. 4:**
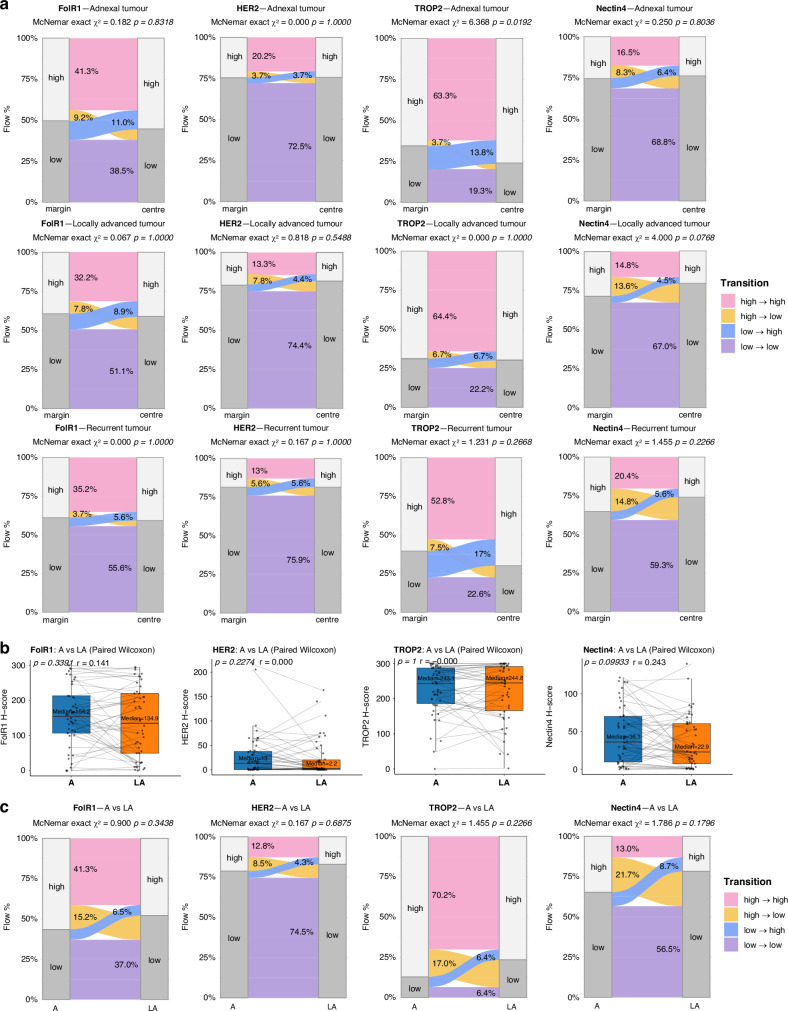
Spatial heterogeneity of ADC target expression in Cohort 2. **a** Sankey plots showing categorical transitions between tumour margin and centre at A, LA, and R across four ADC markers: FolR1, HER2, TROP2, and Nectin-4 (left to right). **b** Paired H-score comparisons between A and LA for the four markers. Statistical significance was assessed using the paired Wilcoxon test. **c** Sankey plots showing categorical group transitions in paired LA and R samples across four ADC markers: FolR1, HER2, TROP2, and Nectin4 (left to right). **Abbreviations**: A adnexal site, LA locally advanced site, R recurrent tumour.

We next analysed centre–margin differences by expression categories. At A, FolR1 showed the highest centre–margin category switching (20.2%) from high (meeting the MIRV treatment-eligibility threshold) to low (not meeting this threshold) expression category or vice versa, followed by TROP2 (17.5%), Nectin-4 (14.7%), and HER2 (7.4%). TROP2 showed a tendency toward higher expression in the tumour centre compared with the tumour margin (McNemar *p* = 0.019; Fig. 4a), whereas FolR1, HER2, and Nectin-4 did not demonstrate significant centre–margin differences (all *p* > 0.8). In LA and R, switching between margin and centre was likewise appreciable: FolR1 16.7% (LA, *p* = 1.000) and 9.3% (R, *p* = 1.000); HER2 12.2% (LA, *p* = 0.549) and 11.2% (R, *p* = 1.000); TROP2 13.4% (LA, *p* = 1.000) and 24.5% (R, *p* = 0.267); Nectin-4 18.1% (LA, *p* = 0.077) and 20.4% (R, *p* = 0.227) (Fig. 4a). Overall, ADC target expression was consistent between margin and centre, and if significant, the effects were inconsistent across cohorts, arguing against a reproducible centre–margin difference.

After evaluating intratumoural centre–margin heterogeneity, we next assessed heterogeneity between matched anatomical tumour sites. Inter-site comparisons between A and LA in Cohort 2 revealed no statistically significant differences in paired H-scores across the four markers (FolR1 *p* = 0.339; HER2 *p* = 0.227; TROP2 *p* = 1.000; Nectin-4 *p* = 0.099; Fig. 4b). Nevertheless, switching between high and low expression categories occurred in a notable fraction of cases: FolR1 showed substantial reclassification (21.7%; *p* = 0.344); switching in the other markers was 12.8% for HER2 (*p* = 0.688), 23.4% for TROP2 (*p* = 0.227), and 30.4% for Nectin-4 (*p* = 0.180) (Fig. 4c).

### ADC targets expression frequently switches between primary and recurrent tumours

To evaluate our hypothesis that ADC targets undergo temporal changes during disease progression, first, we compared A with matched R.

**Fig. 5:**
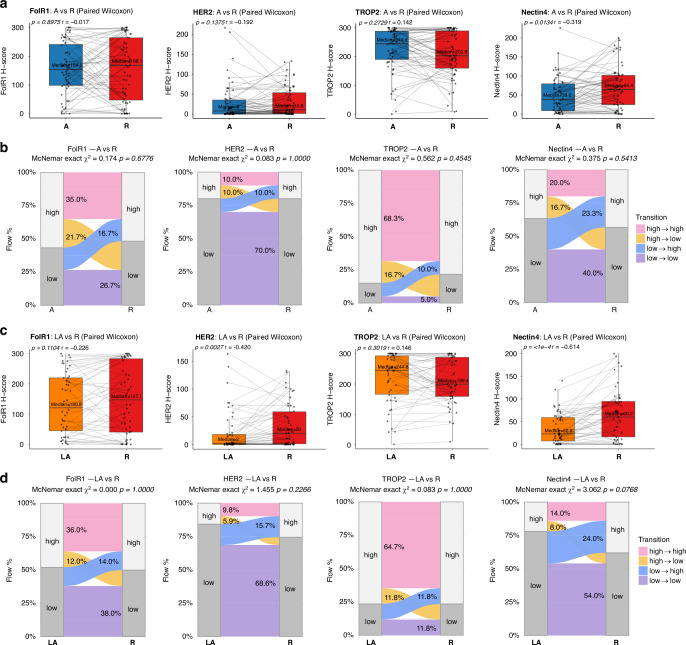
Temporal heterogeneity of ADC target expression in Cohort 2. **a** Paired H-score comparisons between A and R samples for FolR1, HER2, TROP2, and Nectin4. Statistical significance was assessed using the paired Wilcoxon test. **b** Sankey plots showing categorical group transitions in paired A and R samples across four ADC markers: FolR1, HER2, TROP2, and Nectin4 (left to right). **c** Paired H-score comparisons between LA and R samples for FolR1, HER2, TROP2, and Nectin4. Statistical significance was assessed using the paired Wilcoxon test. **d** Sankey plots showing categorical group transitions in paired LA and R samples across four ADC markers: FolR1, HER2, TROP2, and Nectin4 (left to right). **Abbreviations**: A adnexal site, LA locally advanced site, R, recurrence.

H-scores increased for Nectin-4 in R (*p* = 0.013; *r* = 0.319), while HER2, TROP2, and FolR1 showed no significant change (*p* = 0.138, 0.228, 0.258, respectively) (Fig. 5a). For binary IHC classifications, switching between therapeutic-indication groups was substantial: FolR1 38.4% (McNemar *p* = 0.678), Nectin-4 40.0% (McNemar *p* = 0.541), TROP2 26.7% (McNemar *p* = 0.454), and HER2 20.0% (McNemar *p* = 1.000) (Fig. 5b).

Second, we compared LA with matched R (Fig. 5c–d). H-scores increased in R for Nectin-4 (*p* < 0.0001; *r* = 0.614) and HER2 (*p* = 0.003; *r* = 0.420), with no significant change for FolR1 or TROP2 (*p* = 0.110 and *p* = 0.302, respectively). For binary IHC classifications, category switching was also substantial: Nectin-4 38.0% (McNemar *p* = 0.077), TROP2 23.6% (*p* = 1.000), FolR1 26.0% (*p* = 1.000), and HER2 15.7% (*p* = 0.227).

We next explored whether systemic therapy exposure between initial at A site and recurrent surgery was associated with categorical changes in ADC target expression (Supplementary Fig. S6A–C). Across all four ADC targets (FolR1, HER2, TROP2, and Nectin-4), no statistically significant association was observed between bevacizumab exposure and the direction of expression change (all Fisher’s exact *p* ≥ 0.3). Similarly, PARPi exposure did not show consistent associations with switching patterns (all *p* ≥ 0.1). Stratification by chemotherapy cycles (1 vs ≥2) likewise revealed no significant relationship with expression transitions for any marker (all *p* > 0.1). Overall, systemic therapy exposure was not significantly associated with ADC target expression switching between A and R samples. Overall, systemic therapy exposure was not significantly associated with ADC target expression switching between adnexal and recurrent samples.

### Prognostic impact of FolR1 expression is site specific

**Fig. 6:**
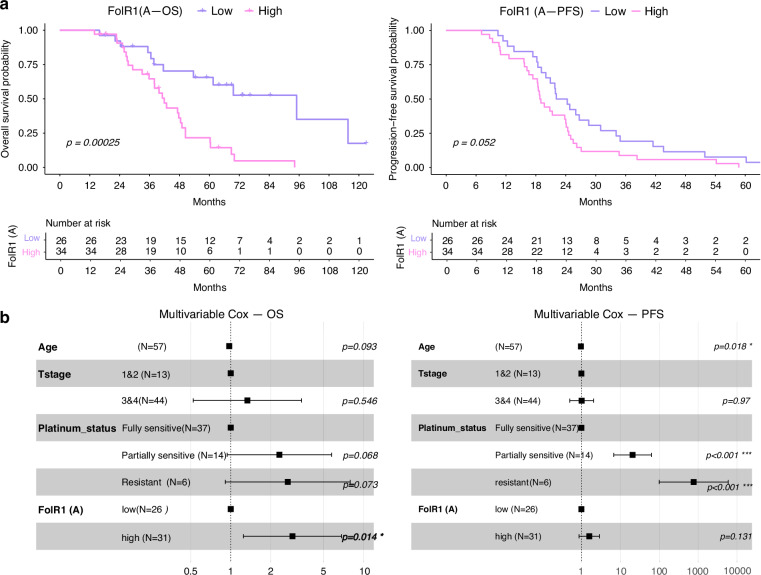
Prognostic impact of FolR1 expression at the adnexal site. **a** Association of FolR1 expression at A site with OS and PFS in Cohort 2 (Kaplan–Meier). **b** Independent prognostic value of FolR1 at A for OS and PFS (multivariable Cox models adjusted for age, T-stage, and platinum sensitivity). **Abbreviations**: A adnexal site, LA locally advanced site, R recurrence, OS overall survival, PFS progression-free survival.

To investigate our hypothesis that ADC marker expression correlates with patient survival, we analyzed the prognostic significance of each marker in Cohort 2, for which we had detailed and long follow-up data. Kaplan–Meier analysis showed that high FolR1 expression in A was significantly associated with worse OS (*p* = 0.0003) and a borderline association with shorter PFS (*p* = 0.052) (Fig. 6a–b). No other ADC markers showed prognostic relevance in A (Supplementary Fig. S7A, B). The expression of FolR1 at LA did not show any association with OS or PFS (Supplementary Fig. S8A—B).

Univariable Cox regression confirmed FolR1 at A as a predictor of poor OS (*p* = 0.001). In multivariable Cox regression adjusting for age, T-stage, and platinum sensitivity, FolR1 expression at A remained an independent predictor of OS (*p* = 0.014), although its association with PFS did not reach significance (*p* = 0.131) (Fig. 6b).

## Discussion

Using two independent TMA cohorts, we comprehensively evaluated molecular, spatial, and temporal heterogeneity of four ADC targets (HER2, TROP2, Nectin-4, FolR1) in HGSOC. We found that: (i) across primary tumours, TROP2 H-scores were lower in *BRCA1/2*-mutated than wild-type tumours (Wilcoxon *p* = 0.029), while no markers showed significant differences by HRD or *TP53*; most cases showed ≥ 2 high-expression targets ; (ii) spatially, there were no generalizable centre–margin or A–LA differences, but the category-switching rates between regions/sites were clinically meaningful, indicating the importance of standardized multi-site and multi-region sampling with IHC; (iii) temporally, Nectin-4 and HER2 tended to be higher at recurrence and FolR1 showed frequent reclassification from A/LA to R, largely independent of prior systemic therapy, underscoring the need for reassessment; and (iv) high FolR1 expression at the adnexal site was associated with worse OS, whereas its expression in recurrent tumours showed no prognostic value.

These findings also provide several integrative implications. At the molecular level, the observation that the majority of cases exhibited more than one high-expression ADC target suggests that patients are not necessarily restricted to a single therapeutic option and that sequential or alternative ADC-targeting strategies may be feasible in clinical practice. Spatially, the absence of consistent centre–margin differences indicates that we found no clear evidence that micro-regional metabolic or immune gradients systematically drive ADC target variability. Moreover, the largely similar expression patterns between A and LA sites are biologically plausible. As both A and LA samples were obtained at the same time point, and adnexal HGSOC frequently already represents disseminated disease rather than the true biological origin, which is increasingly attributed to STICs. Temporally, the observed transitions in ADC target expression between A and R tumours appeared largely independent of prior systemic therapy exposure, including the number of chemotherapy cycles, bevacizumab, and PARPi, suggesting that therapy-related pressure was unlikely to be a major driver of ADC target switching within this cohort.

FolR1 was frequently high at baseline (~56%) and showed no molecular heterogeneity. This prevalence appears higher than the 35–40% reported in MIRV trials and may partly reflect differences in cohort composition [51], as our study only included HGSOC. Consistent with this, Lawson et al. reported that FolR1 positivity was higher in HGSOC (54.3%), while several other subtypes showed minimal or no expression [52]. Likewise, Previs et al. demonstrated that approximately 51% HGSOC were FolR1-positive [51]^.^ Spatially, FolR1 showed the most marked heterogeneity at A, with 20.2% centre–margin reclassification and also showed notable reclassification between paired A and LA (21.7%). The binary IHC categories of FolR1 exhibit a high transition rate over time, 38.4% between A and R, and 26% between LA and R—indicating temporal instability. This further underscores the importance of assessing FolR1 expression in the current biopsy, selecting the progressive lesion as the optimal target for sampling. Prior work by Köbel et al. reported an early OS benefit using a broader positivity definition in primary debulking specimens [53]; our stricter cutoff (≥ 75% moderate/strong) and site-specific analyses (A, LA, R) likely explain the discrepancy. Critically, in our cohort, high FolR1 at A was associated with worse OS and PFS, whereas FolR1 in LA and R was not prognostic. Overall, FolR1 marks baseline aggressiveness and remains a therapeutically relevant target, consistent with the development of MIRV for FolR1-positive platinum-resistant disease [22, 25].

HER2 is largely low in HGSOC, consistent with the historically low rates of HER2 overexpression or amplification [30, 54, 55]. In our cohorts, HER2 expression is not associated with HRD, *BRCA1/2*, or *TP53* mutation type. Spatially, HER2 showed limited switching: margin–centre reclassification was 7.4% at A, 12.2% at LA, and 11.2% at R. Between paired A and LA, only 12.8% of cases changed category. Temporally, HER2 increased at R versus LA (paired Wilcoxon *p* = 0.003, *r* = 0.420), with binary reclassification in 20.0% cases between A and R and 21.6% between LA and R. Consistently, no association with OS or PFS was observed, in line with Luo et al.’s meta-analysis in serous ovarian cancer [30].

TROP2 showed the highest prevalence among the ADC targets (~75–85%). Molecularly, a modest *BRCA1/2*-related decrease in TROP2 H-scores was observed in mutated tumours. Spatially, TROP2 displayed moderate heterogeneity, with 17.5% centre–margin switching at A, 24.5% at R, and 23.4% reclassification between paired A and LA. Temporally, binary categories changed in 26.7% (A–R) and 23.6% (LA–R). Although TROP2 expression was not associated with OS/PFS, its high prevalence across sites and time points indicates reliable target availability, supporting TROP2 as a therapeutic target—in line with the clinical activity of datopotamab deruxtecan (Dato-DXd) in ovarian cancer studies [27–29].

Nectin-4 showed intermediate prevalence in HGSOC (~36–37%) and no association with HRD, *BRCA1/2*, or *TP53* mutation type. Spatially, Nectin-4 displayed appreciable heterogeneity, with 14.7% centre–margin switching at A and 30.4% reclassification between paired A and LA—the highest among the four markers. Temporally, Nectin-4 H-scores increased at recurrence and showed frequent binary reclassification (40.0% between A and R; 32% between LA and R). Taken together, Nectin-4 behaves as a dynamic, site- and time-dependent marker in HGSOC. To our knowledge, this is the first systematic evaluation of Nectin-4 in HGSOC, analyzed alongside other ADC targets. Previous studies have not clearly defined its expression or clinical relevance in this tumour type. Given the clinical success of Nectin-4–directed ADC therapy in urothelial carcinoma, although not prognostic in our cohort, its substantial spatial/temporal instability suggests that Nectin-4 may represent a dynamic and therapeutically tractable target in the R setting of HGSOC.

FDA approval for ovarian cancer therapies often depends on demonstrating a survival benefit. Our data suggests that ADC target expression may have prognostic value, particularly in the primary tumour setting. Similar to *BRCA1/2* as predictive biomarkers for PARP inhibitors, ADC antigen expression might be key to achieving OS benefits. This supports the need for mandatory biomarker-based stratification in ADC trials, ideally through a prospectively defined hierarchical statistical framework.

Our study has several limitations that need to be acknowledged. This study is retrospective and based on TMAs; although each case includes multiple sampling spots, TMAs may still miss subtle spatial heterogeneity observable only in whole-slide images or detectable by spatial omics techniques [20]. We aimed to address this by collecting two regions per tumour — two cores from the centre and two from the margin. This approach has been shown to yield results comparable to whole-slide analysis [56]. Our binary high/low classifications relied on predefined cut-offs, and switching rates are inherently dependent on these thresholds as well as on inter-observer and assay variability. For certain markers, such as Nectin-4, the applied cut-offs were adapted from studies in other tumour types and lack ovarian-specific validation. Importantly, immunohistochemistry-based eligibility criteria are often drug-specific rather than purely target-specific, as different ADCs directed against the same antigen may require distinct scoring systems and clinical thresholds. Further drug-specific and tumour-specific studies are therefore needed to determine and validate optimal predictive biomarker parameters in ovarian cancer. Temporal comparisons may also be influenced by interval length and treatments received between A/LA and R. Sample size limitations reduced the statistical power of certain analyses. Our results support standardized multi-site/multi-region sampling and repeat IHC at recurrence and call for multicentre prospective validation with ovarian-specific cut-offs. However, we acknowledge that multi-site or multi-regional sampling at recurrence is not feasible for the majority of patients who do not undergo secondary cytoreductive surgery, which limits the direct applicability of this strategy in routine clinical practice. When only a single biopsy is obtainable, sampling the biologically most progressive or clinically dominant lesion represents a pragmatic strategy. In cases of discordant multi-site expression, treatment decisions may alternatively be guided by the highest target expression site. However, these approaches remain unvalidated and require prospective confirmation. Furthermore, the potential contribution of the bystander effect may also partially mitigate the impact of inter-site heterogeneity on therapeutic efficacy. Importantly, our study only included patients who were ADC-naïve. As a result, we cannot fully assess how prior exposure to ADCs would alter PFS or OS, nor can we determine how repeated targeting of the same antigen might change the predictive or prognostic value of that antigen over time. This needs to be addressed in future cohorts that include pretreated patients.

Emerging evidence suggests that serum-based markers such as soluble FolR1 may reflect tumour burden and treatment response in ovarian cancer [57]^.^ However, cell-surface protein expression on tumour tissue appears more directly relevant for optimal ADC activity. Therefore, circulating biomarkers are promising for disease monitoring and patient stratification but are unlikely to fully replace tissue-based protein assessment in the context of ADC eligibility. Beyond the ADC targets investigated in this study, emerging targets such as CDH6, B7-H4, and B7-H3 are currently being explored in ovarian cancer and may further broaden ADC-based therapeutic options [42, 43]. Importantly, our findings highlight that spatial and temporal heterogeneity can substantially influence target expression classification, suggesting that similar heterogeneity assessments may be required when evaluating these emerging ADC targets in future research.

ADC targets in HGSOC show limited molecular but marked spatial and temporal heterogeneity. Molecularly, TROP2 H-scores were lower in *BRCA1/2*-mutated cases. No associations were observed among targets or between targets and HRD or *TP53*. The high expression of multiple targets in most cases suggests the feasibility of sequential or alternative targeting strategies. Across targets, we observed clinically meaningful categories switching between regions (margin versus centre) and sites (A versus LA) and, notably, from A/LA to R. These dynamics indicate that a binary IHC call can change with where and when tissue is sampled. Clinically, this supports standardized multi-site and multi-region sampling, explicit site/region documentation, and repeat IHC at recurrence to minimize misclassification and optimize selection for ADC therapy. In addition, FolR1 at the ovarian primary site marks biologically aggressive disease and retains prognostic value (worse OS), helping identify patients who may need closer surveillance and informing trial enrollment or treatment choices at diagnosis. Prospective studies should establish ovarian-specific, drug-specific, practical cut-offs and harmonized sampling standards to improve patient selection for ADCs in HGSOC.

## Supplementary information

Supplementary Material

REMARK-checklist

## Data Availability

Data underlying the results presented in this paper are not publicly available at this time but may be obtained upon reasonable request to MPD (mihnea.dragomir@charite.de).
