# Supplementary material for: Spatial, temporal, and molecular heterogeneity of ADC targets in high-grade serous ovarian carcinoma
Source: Br J Cancer. 2026 Jun 2;135(5):717–27. doi: 10.1038/s41416-026-03482-2 (PMC13478159; doi:10.1038/s41416-026-03482-2)
Supplement: Supplementary file 1 [file 41416_2026_3482_MOESM1_ESM.docx]

**Supplementary material: Spatial, Temporal, and Molecular Heterogeneity of ADC targets in High-Grade Serous Ovarian Carcinoma**

*Xiaoxuan Li et al.*

Supplementary Tables	Page 2-4

Supplementary Figures	Page 5-12

**Supplementary Table S1.** Clinicopathological characteristics and ADC target expression of two cohorts.

| **Variable** | **Cohort 1 (n = 100)** | **Cohort 2 (n = 64)** |
| --- | --- | --- |
| **Age** |  |  |
| <50, n (%) | 15 (15.0) | 19 (29.7) |
| ≥50, n (%) | 85 (85.0) | 45 (70.3) |
| median [IQR] | 62 [54–72] | 54 [48.8–63.3] |
| **FIGO stage, n (%)** |  |  |
| I–II | 7 (8.6) | 6 (10.3) |
| III–IV | 74 (91.4) | 52 (89.7) |
| **ADC marker expression in primary ovarian tumor** | | |
| **Qualitative (High expression, n (%))** |  |  |
| HER2 | 34 (34.0) | 12 (20.0) |
| Nectin4 | 36 (36.0) | 22 (36.7) |
| TROP2 | 75 (75.0) | 51 (85.0) |
| FolR1 | 56 (56.0) | 34 (56.7) |
| **Quantitative (H-score, median [IQR])** |  |  |
| HER2 | 30.0 [5.4–82.5] | 8.0 [0.3, 35.6] |
| Nectin4 | 51.5 [19.1–89.0] | 38.6 [9.0, 79.4] |
| TROP2 | 207.0 [155.3–236.3] | 244.4[190.3, 287.8] |
| FolR1 | 176.3 [106.3–238.0] | 154.25 [98.7 – 240.6] |
| **Follow-up** |  |  |
| Complete follow-up, n (%) | 93 (93.0) | 64 (100.0) |
| Follow-up time (months, median [IQR]) | 17.3 [11.1–21.5] | 41.7 [28.8–63.7] |
| Recurrence, n (%) | 10 (10.8) | 64 (100.0) |
| Death, n (%) | 14 (15.1) | 42 (65.6) |

**Abbreviations:** ADC, antibody–drug conjugate; IQR, interquartile range; FIGO, International Federation of Gynecology and Obstetrics; H-score, histological score; HER2, human epidermal growth factor receptor 2; TROP2, trophoblast cell surface antigen 2; FolR1, folate receptor alpha.

**Supplementary Table S2.** Systemic therapies administered between primary and recurrent surgery in Cohort 2.

| **Treatment before recurrence** | **Subgroup** | **N (%)** |
| --- | --- | --- |
| **Bevacizumab** | Yes | 51 (79.7%) |
|  | No | 13 (20.3%) |
|  |  |  |
| **PARPi** | Yes | 36 (56.3%) |
|  | No | 28 (43.7%) |
|  |  |  |
| **Chemotherapy** | 1 cycle | 55 (85.9%) |
|  | ≥2 cycles | 9 (14.1%) |

Abbreviations: PARPi, Poly (ADP-ribose) polymerase inhibitor.

**Supplementary Figure S1. Definition and representative examples of tumour centre and margin.**

**(A)** The selection of tumour centre and tumour margin regions within the same paraffin block. Scale bar = 2 mm. **(B)** Representative H&E-stained TMA cores from tumour centre and tumour margin regions. Scale bar = 1 mm.

**Supplementary Figure S2. Associations of ADC target expression with genomic features in Cohort 1.**

**(A)** Comparisons between HRD groups (NEG vs POS) for FolR1, HER2, TROP2, and Nectin-4 H-scores.

**(B)** Comparisons between *BRCA1/2* groups (wild type vs mutated) for FolR1, HER2, TROP2, and Nectin-4 H-scores. **(C)** Comparisons between *TP53* mutation classes (in-frame/missense vs splice-site/truncating) for FolR1, HER2, TROP2, and Nectin-4 H-scores.

**Abbreviations**: HRD, homologous recombination deficiency; NEG, negative; POS, positive.

**Supplementary Figure S3. Associations of ADC target expression level with *BRCA1/2* status in Cohort 1.**

Proportion of ADC target expression groups stratified by *BRCA1/2* status (wild type vs mutated) for FolR1, HER2, TROP2, and Nectin-4.

**Supplementary Figure S4. Pairwise correlations among ADC targets in Cohort 1.**

Scatterplots of all pairwise combinations of FolR1, HER2, TROP2, and Nectin-4 H-scores. Each point represents one tumor; the blue line shows a least-squares fit.

**Supplementary Figure S5. Margin–centre comparisons at A, LA, and R across four ADC markers. The analysis of adnexal tumours and locally advanced tumours is performed in both cohorts combined.**

**(A)** Paired H-score comparisons of margin versus centre for FolR1, HER2, TROP2, and Nectin-4 at A. **(B)** Paired H-score comparisons of margin versus centre for FolR1, HER2, TROP2, and Nectin-4 at LA. **(C)** Paired H-score comparisons of margin versus centre for FolR1, HER2, TROP2, and Nectin-4 at R. **Abbreviations**: A, Adnexal site; LA, Locally advanced site; R, recurrent site.

**Supplementary Figure S6. Association between systemic therapy exposure and temporal changes in ADC target expression in Cohort 2.**

**(A)** Categorical transitions in ADC target expression between A and R stratified by bevacizumab therapy for FolR1, HER2, TROP2, and Nectin-4. **(B)** Categorical transitions in ADC target expression between A and R stratified by PARPi therapy for FolR1, HER2, TROP2, and Nectin-4. **(C)** Categorical transitions in ADC target expression between A and R stratified by chemotherapy cycles for FolR1, HER2, TROP2, and Nectin-4.

**Abbreviations:** A, adnexal site; R, recurrent site; PARPi, Poly (ADP-ribose) polymerase inhibitor.

**Supplementary Figure S7. Prognostic analyses at the A site in Cohort 2.**

**(A)** Kaplan–Meier curves comparing OS between high vs low expression groups for HER2, TROP2, and Nectin-4 in A tumours. **(B)** Kaplan–Meier curves comparing PFS between high vs low expression groups for HER2, TROP2, and Nectin-4 in A tumours.

**Abbreviations**: A, adnexal site; OS, overall survival; PFS, progression-free survival.

**Supplementary Figure S8. Prognostic analyses at LA and R site in Cohort 2.**

**(A)** Kaplan–Meier curves comparing OS between high vs low expression groups for FolR1, HER2, TROP2, and Nectin-4 in LA tumours. **(B)** Kaplan–Meier curves comparing PFS between high vs low expression groups for FolR1, HER2, TROP2, and Nectin-4 in LA tumours. **(C)** Kaplan–Meier curves comparing PRS between high vs low expression groups for HER2, TROP2, and Nectin-4 in PD tumors.

**Abbreviations**: LA, locally advanced site; R, recurrent site; OS, overall survival; PFS, progression-free survival.
